# Mesoporous Composite Bioactive Compound Delivery System for Wound-Healing Processes

**DOI:** 10.3390/pharmaceutics15092258

**Published:** 2023-08-31

**Authors:** Bogdan Purcăreanu, Manuela Diana Ene, Alina Moroșan, Dan Eduard Mihaiescu, Mihai Alexandru Florea, Adelina Ghica, Roxana Andreea Nita, Veronica Drumea, Mihai Alexandru Grigoroscuta, Andrei Kuncser, Petre Badica, Laura Olariu

**Affiliations:** 1 Biotehnos SA, Gorunului Street 3-5, 075100 Otopeni, Romania or bogdanpb89@gmail.com (B.P.); alexandru.florea@biotehnos.com (M.A.F.); adelina.bicu@biotehnos.com (A.G.); rnita@biotehnos.com (R.A.N.); veronica.drumea@biotehnos.com (V.D.); lolariu@biotehnos.com (L.O.); 2 Department of Science and Engineering of Oxide Materials and Nanomaterials, Faculty of Chemical Engineering and Biotechnologies, University Politehnica of Bucharest, Gh. Polizu 1-7, 011061 Bucharest, Romania; 3 Department of Organic Chemistry “Costin Neniţescu”, Faculty of Chemical Engineering and Biotechnologies, University POLITEHNICA of Bucharest, Gh. Polizu 1-7, 011061 Bucharest, Romania; alina.morosan@upb.ro; 4 National Institute of Materials Physics, Street Atomistilor 405 A, 077125 Magurele, Romania; alex_bebe07@yahoo.com (M.A.G.); akuncser@yahoo.com (A.K.); badica2003@yahoo.com (P.B.); 5 Academy of Romanian Scientists, 3 Ilfov Street, 030167, Bucharest, Romania

**Keywords:** MCM-41, mesoporous material, composites, bioactive extract, biological activity, wound healing

## Abstract

Currently, the treatment of wounds is still a challenge for healthcare professionals due to high complication incidences and social impacts, and the development of biocompatible and efficient medicines remains a goal. In this regard, mesoporous materials loaded with bioactive compounds from natural extracts have a high potential for wound treatment due to their nontoxicity, high loading capacity and slow drug release. MCM-41-type mesoporous material was synthesized by using sodium trisilicate as a silica source at room temperature and normal pressure. The synthesized mesoporous silica was characterized by using Scanning Electron Microscopy (SEM), Transmission Electron Microscopy (TEM), N_2_ absorption–desorption (BET), Dynamic Light Scattering (DLS) and Fourier transform infrared spectroscopy (FT-IR), revealing a high surface area (BET, 1244 m^2^/g); pore diameter of approx. 2 nm; and a homogenous, ordered and hexagonal geometry (TEM images). Qualitative monitoring of the desorption degree of the *Salvia officinalis* (SO) extract, rich in ursolic acid and oleanolic acid, and *Calendula officinalis* (CO) extract, rich in polyphenols and flavones, was performed via the continuous recording of the UV-VIS spectra at predetermined intervals. The active ingredients in the new composite MCM-41/sage and marigold (MCM-41/SO&CO) were quantified by using HPLC-DAD and LC-MS-MS techniques. The evaluation of the biological composites’ activity on the wound site was performed on two cell lines, HS27 and HaCaT, naturally involved in tissue-regeneration processes. The experimental results revealed the ability to stimulate collagen biosynthesis, the enzymatic activity of the main metalloproteinases (MMP-2 and MMP-9) involved in tissue remodeling processes and the migration rate in the wound site, thus providing insights into the re-epithelializing properties of mesoporous composites.

## 1. Introduction

The prevalence and high degree of complications that can occur when the integrity of the skin is compromised is one of the major concerns in health care, and it becomes more challenging when chronic wounds are associated with other pathologies that can disrupt the healing process (diabetes mellitus, systemic atherosclerosis or local ischemia and pressure ulcers) [1,2,3,4,5,6]. In general, all wounds tend to be chronic and are characterized by prolonged inflammation and senescent, immature or highly differentiated fibroblasts that respond ineffectively to the stimulation of the cytokines involved in the healing process [7,8,9,10,11,12,13]. In response to injury, histamines, free radicals and inflammatory cytokines are released, and they amplify vasodilatory processes and tissue edema [12,14,15,16,17], promoting the penetration of inflammatory cells into the wound site, activating the cascade of events which leads to the elimination of cellular and tissue debris, releasing growth factors and initiating the reorganization of the extracellular matrix [18,19,20] by activating metalloproteinases that affect the intercellular matrix, arresting the wound at the inflammatory stage [18,19,20,21,22]. The chemotactic factors released by the inflammatory cells induce the population of the wound with fibroblasts that migrate into the fibrin scaffold, increasing collagen synthesis [4,23,24,25]. Epithelial cells on the basement membrane increase in size, emit pseudopodia and migrate into the wound site [18,23,24,25]. The reepithelization of the wound by keratinocytes follows, and vascularized granular tissue is replenished by endothelial and fibroblast cells, which leads to the recovery of the elasticity of injured tissue [26,27,28]. Taking into account the need to diversify therapeutic approaches, new R&D strategies exploring emerging technologies have been initiated to achieve smart therapeutic solutions that can ensure an efficient healing process. The pharmaceutical industry is directed towards the development of biocompatible products, with as few adverse reactions as possible, with the goal of obtaining high compliance from patients and favorable results in the healing process [16]. Natural bioactive extracts and plant-derived substances have shown significant promise due to their complex composition and phytotherapeutically activity, with great potential to enhance the wound-healing process through different paths [29]. The literature data highlight the in vitro antioxidant and antimicrobial activity of sage extracts and ursolic and oleanolic acid, showing that they are effective against *Streptococcus pneumoniae* and methicillin-resistant *Staphylococcus aureus* (MRSA) strains. Also, there is evidence that attributes an anti-inflammatory effect to ursolic acid, which was found to have twice the potency of indomethacin [30]. The results of a study showed that hydroethanolic leaf extract increased wound contraction, associated with re-epithelization, and increased new vessel formation with a fibroblast distribution [30,31]. Even sage essential oil, through its cis- and trans- thujone, camphor and 1,8-cineole content, shortened the inflammatory phase, with the acceleration of cellular proliferation and revascularization, collagen deposition and re-epithelization [32]. Marigold extract is well known to have many benefits in the wound-remodeling process due to its anti-inflammatory, antiedematous and antibacterial efficacy and due to its capacity to enhance neovascularization (stimulating angiogenesis) at the wound site, promoting the healing process [29,33,34].

Starting with the major benefits of the use of biologically active compounds of natural origin in the treatment of chronic wounds, advantageous technical solutions were sought for matrix-type materials and encapsulation alternatives, which would allow for an adsorption ratio as high as possible and, at the same time, a modified release profile of the compounds of interest, ensuring sustained action. The proposed solution involves the use of MCM-41 as a matrix, with its high load capacity, due to its specific surface area exceeding 1000 m^2^/g, pore volume > 0.6 cm^3^ and pore diameter of 2–4 nm. There were several reasons behind this approach: the stability of calendula and sage hydroalcoholic extracts (similar for other plants) is known to be uncertain in terms of medium- to long-term preservation, involving different technical solutions related to the several additives used (which certainly generate concerns and side effects); this issue also involves analytical problems for the final product and interphase testing and quantification. The mesoporous coating provides a stable solid matrix (easily identified and quantified by MID/NIR or a Raman analysis by using classification methods, i.e., PCR/PLS for the control of the final product and critical points in the workflow) with significantly increased stability and a reduced release capacity (a real clue for wound-healing applications). Due to this behavior, the proposed materials offer new insight into wound-healing applications related to subsequent biopolymer encapsulation, bioprinting applications, regenerative treatments and the stabilization of plant extracts.

Mesoporous silica has attracted the attention of researchers in the field ever since its discovery by the Mobil Corporation due to its special properties, high specific surface area, large pore volume, easy functionalization and high biocompatibility. Generally, mesoporous silica can be easily obtained by using a cationic surfactant as a template, which serves as a structure-directing agent for silica polycondensation (a silica source) through electrostatic interactions [35,36,37,38,39].

The goal of the present study was to synthesize a mesoporous MCM-41-type material at room temperature and normal pressure by using sodium trisilicate as a cheaper alternative to tetraethyl orthosilicate (TEOS) as a carrier for the bioactive compounds in the extracts of *Salvia officinalis* and *Calendula officinalis* for wound-healing dressing. The mesoporous material obtained was purified by using ethanolic solution extraction; then, it was dried and finally calcined at 570 °C to completely remove the surfactant used to shape and size the pores.

After obtaining the material, a BET analysis was performed to determine the specific surface area, volume and diameter and microscopy techniques (SEM and TEM) to investigate the morphology of the mesoporous silica. Infrared spectroscopy was used to highlight the removal of CTAB from the pores and the loading of the calcined MCM-41-type material with bioactive compounds from plant extracts.

To evaluate the potential therapeutic effect of the management of tegumentary wounds, experimental models were developed and implemented on epidermal-specific cell lines (fibroblasts and keratinocytes), and we recorded cellular effusion in the presence of delivery systems (MCM-41/CO, MCM-41/SO and MCM-41/SO&CO) for the main biomolecules involved in tissue regeneration (collagen and metalloproteinases) and the migration velocity into the wound site under normal growth conditions or the induction of oxidative stress with PMA and TNF-α.

## 2. Materials and Methods

### 2.1. Materials and Equipment

Sodium trisilicate (Na_2_Si_3_O_7_, Santa Cruz Biotechnology, Dallas, TX, USA) as a silica source and Hexadecyltrimethylammonium bromide (CTAB; C_19_H_42_BrN, ≥99%, Sigma-Aldrich, Saint Louis, MI, USA) as a template (a structure-directing agent) were used for the synthesis of the MCM-41 material. Sodium hydroxide pellets (NaOH; >98%, Bernd Kraft, Duisburg, Germany) were used to ensure the alkaline pH necessary to obtain the trisilicate solution. An ammonium hydroxide solution (NH_4_OH; puriss. p.a., 25% NH_3_ basis, Honeywell Research Chemicals, Germany), glacial acetic acid (C_2_H_4_O_2_, Honeywell Research Chemicals, Germany) and ethanol (C_2_H_6_O; ≥99.8%, Honeywell Research Chemicals) were used as reagents when obtaining the two solutions (acid and alkaline) subjected to precipitation. Ultrapure water from the Millipore Integral 3 water purification system was used. Methanol, RS for LC/MS (CH_4_O; Carlo Erba Reagent GMBH, Germany) and o-phosphoric acid (H_3_PO_4_; 85%, Merck, Germany) were used for the mobile phase in the HPLC–DAD analysis. Methanol and acetic acid for LC/MS (C_2_H_4_O_2_; 100%, Merck) were used for the mobile phase in the LC-MS-MS analysis. As is standard when using the quantification method, we used chlorogenic acid (C_16_H_18_O_9_; ≥95%, Santa Cruz Biotechnology), hyperoside, the Primary Reference Standard (C_21_H_20_O_12_; 95.9%, HWI Group, Germany), oleanolic acid (C_30_H_48_O_3_; ≥97%, Santa Cruz Biotechnology) and ursolic acid (C_30_H_48_O_3_; ≥90%, Sigma-Aldrich). To establish the biological action of the composite materials, standardized cell lines specific to the dermoepidermal layer were used as follows: human dermal fibroblasts (normal cell line HS27—ATCC^®^ CRL-1634 ™) cultivated in Dulbecco’s Modified Eagle’s Medium/Nutrient Mixture F-12 Ham (Sigma-Aldrich) supplemented with 10% fetal bovine serum (Sigma-Aldrich) and 1% Antibiotic–Antimycotic Solution (100×) (Sigma-Aldrich) and normal human keratinocytes (immortalized cell line HaCaT purchased from ThermoFisher Scientific, Walthan, MA, USA) cultured in Dulbecco’s Modified Eagle’s Medium (ATCC^®^) with high content of glucose supplemented with 10% fetal bovine serum and 1% Antibiotic–Antimycotic Solution (100×) purchased from Sigma-Aldrich. Chemical reagents of analytical grade like 0.010 M phosphate-buffered saline, pH = 7.4; phorbol myristate acetate; TNF-α; hydrochloric acid; chloramine T; propyl alcohol; p-dimethylaminobenzaldehyde; and hydroxyproline were purchased from Sigma-Aldrich.

In the material-purification process, we used a Memmert GmbH UF110 oven (Schwabach, Germany), a Protherm ECO15/110 chamber furnace (Ankara, Turkey) and a Retsch PM100 planetary ball mill (Haan, Germany).

The loading process was performed in a Buchi rotary evaporator R-300 (Flawil, Switzerland), and we accurately weighed the materials with the help of the Mettler Toledo Excellence XS 205 DU/M analytical balance (Schweiz, Switzerland).

For the characterization of MCM-41, we used a Scanning Electron Microscope Lyra3XMU with a Focused Ion Beam SEM-FIB sample-preparation system (Tescan, Brno-Kohoutovice, Czech Republic) with an acceleration voltage of 200 V and an SE resolution 1.2 nm at 30 kV in High Vacuum mode; JEOL JEM-2100 high-resolution analytical Transmission Electron Microscope (TEM) with an acceleration voltage of 200 kV; Malvern Nano ZS Zetasizer instrument (Dynamic Light Scattering, DLS) (Malvern, UK); Anton Paar Physisorption Analyzer Nova 800 (USA); and Thermo Scientific Nicolet 6700 FT-IR-ATR spectrometer with a zinc selenium crystal (SnZn) (Waltham, MA, USA).

The qualitative monitoring of the profile desorption was carried out with a Thermo Scientific Evolution 220 UV-VIS spectrophotometer (Waltham, MA, USA). For the quantification of the loaded active principles and to obtain release profiles, we used the Agilent 1260 series HPLC—DAD system (Agilent, Santa Clara, CA, USA), and we used the Agilent 1200 series HPLC coupled with the Agilent TripleQuad 6410 Mass Spectrometer for the phenolic acids analysis and triterpenic acids analysis.

The cell lines were maintained in a culture in a Steri-Cycle I160 CO_2_ incubator from Thermo Scientific, and the culture evolution was monitored by using a NiKon Eclipse Ti reverse-phase microscope equipped with an incubation chamber and a recording camera. An absorbance recording was carried out by using a PerkinElmerLambda 25 UV-VIS spectrophotometer, and electrophoretic analyses were performed with a Mini-PROTEAN Tetra System (Bio-Rad Laboratories S.r.l., Milano, Italy).

### 2.2. Methods

#### 2.2.1. Synthesis of Mesoporous MCM-41-Type Material

MCM-41 mesoporous silica was synthesized at an ambient temperature in an alkaline medium by using the sol-gel method. Sodium hydroxide (0.64 M) was dissolved in ultrapure water under a constant temperature, and then sodium trisilicate (0.5 M) was added with stirring. After obtaining the trisilicate solution, the CTAB (0.05 M) was added and stirred until complete dissolution. The final reaction (the precipitation phenomenon) occurred when the alkaline solution interacted with the acid acetic solution (2.1 M). The precipitated material was obtained through filtration and dried at 105 °C for a minimum of 12 h in the oven. In order to remove the surfactant from the pores, the material was submitted to a purification process by applying 3 consecutive stages of extraction with a specific amount of a 90% ethanolic solution, grinding at the ball mill and calcination at 570 °C for 12 h in the furnace. At the end of the process, a white MCM-41 powder was obtained.

#### 2.2.2. Characterization of Mesoporous MCM-41-Type Material

The textural properties of the resulting material were characterized by using both classical methods and advanced spectrometric and imaging methods. The morphology of the samples was investigated by using Scanning Electron Microscopy (SEM), providing information about the surface morphology, and Transmission Electron Microscopy (TEM), providing details about the pores’ shape and size. The processing stage of the powder samples was performed on aluminum stubs with double-sided carbon stickers for the SEM observation and on a carbon-plated copper grid (the standard powder method) for the TEM observation.

The average hydrodynamic particle diameter was determined by using the Dynamic Light Scattering (DLS) method. The samples were dispersed in ultrapure water and sonicated for 5 min and then measured in dedicated cuvettes (DTS0012).

Nitrogen adsorption–desorption curves of the samples were obtained at 77 K (−196.15 °C). Prior to the analysis, the samples were outgassed under vacuum at 300 °C for 4 h from the adsorption branch of the isotherm; we used the Brunauer–Emmett–Teller (B.E.T.) method to calculate the specific surface area, and from the desorption branch, we used the Barrett–Joyner–Halenda (B.J.H.) method to estimate the pore diameter distribution. Also, the type of isotherm can indicate the MCM-41 inclusion in a certain class of materials, which are classified based on the isotherm.

Using Fourier transform infrared spectroscopy (FT-IR), we recorded the MID-infrared (4000 to 550 cm^−1^) spectra of the powder samples, which make up the “spectral fingerprint”. For loaded samples, a spectrometric analysis can provide proof of the loading process by highlighting the specific functional groups of the bioactive extracts. Approximately 5–10 mg of powder was placed on the surface of the SeZn crystal of the ATR, and then it was pressed gently and analyzed directly. A background spectrum was collected for each sample. The infrared spectrum of the sample was collected at an 8 cm^−1^ resolution, with a reading interval of 4 cm^−1^ and 32 scans. The same parameters were applied to the background spectrum as well.

#### 2.2.3. Loading Process of Bioactive Extracts on Mesoporous Matrix 

*Salvia officinalis* (SO) extract was obtained by the cold maceration of ground sage leaves by using 70% ethanol as the extraction solvent for 24 h. To remove the plant debris, the hydroalcoholic solution was passed through a paper filter. To remove the chlorophyll pigments, purification with activated charcoal was achieved. *Calendula officinalis* extract (CO) was obtained by the cold maceration of marigold flowers, using 65% ethanol as the extraction solvent, for 24 h. For the removal of plant residues, the hydroalcoholic solution was passed through a paper filter.

The MCM-41 was loaded with 30% (*w*/*w*) bioactive extracts (*Salvia officinalis* (SO) extract, rich in ursolic acid and oleanolic acid, and *Calendula officinalis* (CO) extract, rich in polyphenols and flavones) by using a gradient concentration technique. It is a simple, efficient and reproducible method involving the gradual evaporation of the solvent by using a rotary evaporator under vacuum and controlled temperature conditions (50 °C) in order to reduce the time required and to avoid the degradation of the organic compounds. The MCM-41-type mesoporous material was introduced into an evaporation flask, over which the hydroalcoholic extract was added. The pressure was gradually increased until the solvent was completely removed. The resulting complexes (MCM-41/SO and MCM-41/CO) were dried at 60 °C in the oven overnight and then stored at room temperature. Furthermore, these two types of loaded materials were used to obtain a combination (MCM-41/SO&CO) with improved therapeutic values due to the synergism of the extracts.

#### 2.2.4. Quantification of Bioactive Compounds and Release Profiles of Composite Materials

Qualitative monitoring of the desorption degree of the whole bioactive complex loaded in the pores of the synthesized composites was performed via the continuous recording of the spectra for a period of time at predetermined intervals by using a UV-VIS spectrophotometer whose reading cell was connected, through a peristaltic pump, to the vessel in which the desorption took place. The weighed samples were encapsulated in filter paper (nacelle) and kept suspended, using a rod system, in a 100 mL medium (60% ethanolic solution, acidified 0.05% with acetic acid) at a constant temperature of 36 °C. The monitoring period was 3 h for the MCM-41/CO and MCM-41/SO composites and 24 h for MCM-41/SO&CO. The recording times at which the monitoring was performed were established according to the therapeutic target. The release profile of the compounds was obtained in a continuous flow in the spectral range of 190–400 nm. The acquisition parameters considered the width of the spectral band (=2 nm), the scanning speed (=300 nm/min), the integration time (=0.1 s) and the number of recording cycles (240).

The active ingredients in the MCM-41/SO&CO composite were quantified by using High-Performance Liquid Chromatography–Diode Array Detector (HPLC-DAD) and Liquid Chromatography—Mass Spectrometer (LC-MS-MS) techniques. The Accurately weighed samples (approx. 100 mg), together with 2 mL of the medium, were placed in plastic dialysis bags (Spectra/Por MWCO 12–14 kDa) and were suspended in 98 mL of the medium, in which the magnetic system ensured a permanent homogenization. A total of 2 mL of the sample was taken at the predetermined time intervals, and the volume was determined after each sampling with the initially prepared medium solution. The samples were then filtered through 0.45 µm PTFE membranes and analyzed chromatographically.

An HPLC analysis of the triterpene acids (oleanolic and ursolic acids) was performed by using the HPLC-DAD system (Agilent 1260 series, Santa Clara, CA, USA) with a reverse-phase C18 analytical column (100 × 4.6 mm, 3 µm particle size) and a mobile phase consisting of methanol and 0.1% phosphoric acid (84:16, *v*/*v*). The UV detector was set at 205 nm, and the flow rate was 0.6 mL/min.

The analysis of the phenolic acids was performed on an Agilent 1200 series LC coupled with an Agilent TripleQuad 6410 Mass Spectrometer equipped with a multimode source. The HPLC separation was carried out on a reverse-phase C18 analytical column (100 mm × 3 mm, 3.5 μm particle size) with a mobile phase consisting of methanol and 5 mM acetic acid (25:75, *v*/*v*) at a flow rate of 0.4 mL/min. The analytes were quantified by Multiple Reaction Monitoring (MRM) in negative electrospray ionization mode. The following transitions were monitored for the target compounds: *m/z* 353 → 191 for chlorogenic acid, *m/z* 179 → 135 for caffeic acid and *m/z* 463 → 300/271 for hyperoside. For the acquisition and postacquisition analysis, Agilent MassHunter software B.06.00 was used. All the samples were quantified by using external standard calibration methods.

#### 2.2.5. Evaluation of the Biological Action of Composite Materials

The investigations were focused on the evaluation of the biological action of mesoporous composites on the migration rate of epithelial cells into the wound site and the modulation of tissue-remodeling processes in terms of collagen biosynthesis and the activity of the matrix proteolytic enzymes MMP-2/MMP-9 (two metalloproteinases with an extremely important role in the physiological processes of tissue remodeling and cell migration) [40]. The experimental system consisted of fibroblast cells (normal cell line HS27) and squamous epithelial keratinocyte cells (cell line HaCaT) and was left to adhere for 24 h at an inoculum of 70,000 cells/well in a 24-well plate; then, it was treated for a period of 72 h with mesoporous silica-based material loaded with plant extracts (MCM-41/SO, MCM-41/CO and MCM-41/SO&CO). This experimental model was composed of a cell control (untreated cells); positive control (Vitamin C—10 µg/mL) and controls corresponding to mesoporous silica-based material without the loaded plant extract (MCM-41). Two experimental series were performed under unstimulated and proinflammatory stimulated conditions with 0.1 mM PMA (phorbol myristate acetate) and 15 ng/mL TNF-α for 24 h. At the end of the cell exposure to the test substances, the culture supernatant was collected for further analysis of the enzymatic activity of the MMP-2 and MMP-9 secreted into the extracellular medium by both the studied lines, and the concentration of the collagen secreted into the extracellular medium by HS27 was determined.

(a) The collagen secreted by the fibroblast cell in the culture medium was assessed indirectly by using a colorimetric method for the determination of hydroxyproline resulting from the total hydrolysis of collagen polypeptide chains [41]. This could be performed because 1 mg of collagen contains 0.122 mg of hydroxyproline. Thus, the hydroxyproline released from the hydrolysis process was oxidized with the oxidizing agent chloramine T to pyrrole, a compound that forms with p-dimethylaminobenzaldehyde (DMAB, Ehrlich’s reagent), a chromophore with an absorption maximum in the range 555–560 nm. The colorimetric determination of hydroxyproline was conducted by using the Woessner method modified in house: the medium samples were subjected to chemical hydrolysis under the action of hydrochloric acid for 6 h at 120 °C. The mixture consisting of a hydrolysate and chloramine T solution (0.352 g of chloramine T, 7.5 mL of propyl alcohol, 5 mL of distilled water and 12.5 mL of citrate buffer, pH 6.0) in a 1:1 (*v*/*v*) ratio was shaken vigorously and kept at room temperature for 20 min. At the end of the standing time, 1 mL of the p-dimethylaminobenzaldehyde solution was added and incubated for 30 min at 60 °C. After the incubation time was completed, the absorbance of the samples was recorded at a wavelength of 560 nm, and the hydroxyproline content was quantified relative to a standard curve generated by using synthetic hydroxyproline [41].
(b) The method for estimating the enzyme activity of the matrix gelatinases (MMP-2 and MMP-9) from the culture media samples was based on the ability of these enzymes to renature after electrophoretic migration in polyacrylamide SDS-PAGE gel copolymerized with 0.1% gelatin under nonreducing conditions run at 120 V in a Tris-glycine buffer, as described by Cancemi et al. [42,43], and the removal of SDS by repeated washes with a 5% Triton X-100 solution. The renatured enzymes exert their proteolytic activity on the copolymerized substrate during 18 h of incubation at 37 °C in a buffer consisting of 50 mM Tris-HCl (pH 7.5), 0.15 M NaCl, 0.01 M CaCl_2_ and 1 µM ZnCl_2_ [43]. The identification of MMPs was performed after staining the gels with 0.25% Coomassie blue R-250, destaining with methanol: acetic acid: water (40:10:50; *v*/*v*) and correlating them with a molecular mass standard. The two digestion zones (corresponding to molecular masses of 92 kDa for MMP-9 and 62 kDa for MMP-2) appear white on a dark blue background. The zymograms were scanned with the ChemiDoc MP Imaging System from BIO-RAD and were semiquantitatively analyzed by using the densitometry of the enzymes’ active protein bands which appear as lysing bands by using Image J software. All the experiments were performed in triplicate. During the electrophoretic migration process, endogenous MMP-specific inhibitors (TIMPs), which are frequently found to be associated with MMPs in the culture medium, dissociate from proteinzyme, thus eliminating negative potential interferences that may affect the experimental results.
(c) To evaluate in vitro the effect of the mesoporous composite systems used for the release of bioactive compounds on the migration rate of HS27 cells into the wound site, an experimental model was applied that required a wound to be simulated in a confluent layer of cells by scratching. In each well, 1 mL of a collagen-containing solution was pipetted. After a one-hour incubation, to allow the protein to adhere to the culture surface, the protein solution was removed and the well was washed three times with 0.010 M phosphate-buffered saline, pH = 7.4. In total, 30,000 cells/well were inoculated into the prepared plate and allowed to adhere for 24 h at 37 °C, 5% CO_2_. Subsequently, by using a pipette tip, the cell monolayer was scraped off at confluence. After this operation, the cell layer was washed, the growth medium was replaced and the substances to be evaluated were introduced, and thus the migration of the cells to the area from which they were removed (wound) and the reformation of the cell layer were monitored.

#### 2.2.6. Statistical Analysis

Statistical processing was performed by using an analysis of variance (ANOVA) (95% significant level) on each pair of interest, and differences at *p* < 0.05 were considered statistically significant. The data were processed by using GraphPad Prism software version 5.0 (GraphPad Software, La Jolla, CA, USA). The statistical analysis was performed by applying the ANOVA method (to indicate a significant difference vs. the cell control).

## 3. Results

### 3.1. Characterization of Mesoporous MCM-41-Type Material

**Figure 1:**
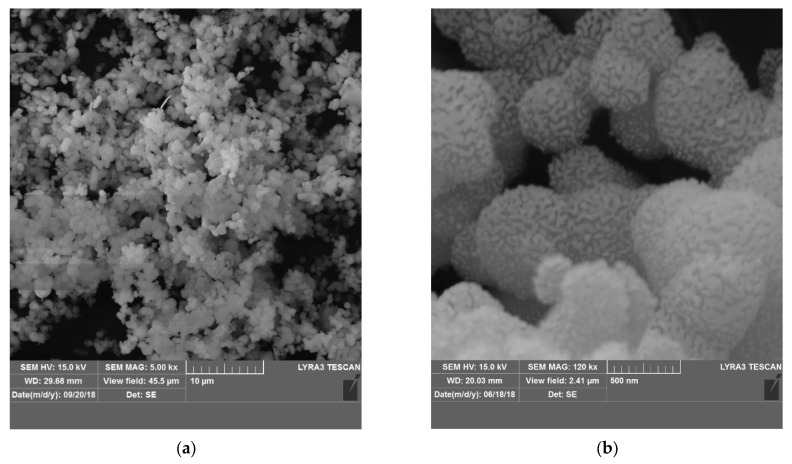

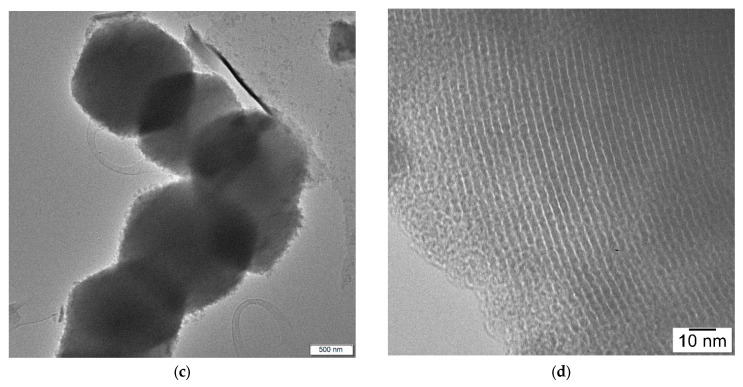
SEM images (**a**,**b**) and TEM images (**c**,**d**) at different magnifications taken of the calcined mesoporous material of type MCM-41.

The microstructure of MCM-41 is presented in SEM images (Figure 1a,b and Supplementary Materials, Figure S1a,b). The material is composed of spherical particles with a size in the range of 0.5–1.5 microns (Figure 1b and Supplementary Materials, Figure S1b). Some particles are not individual and form complex shape formations with a larger diameter between 1–6 microns (Figure 1a,b and Supplementary Materials, Figure S1b). In some cases, the shape deviates and the appearance has features of a polyhedron where angles about 120° can be distinguished with difficulty (Figure 1b,c). Particles and formations show a porous surface (Figure 1b,d and Supplementary Materials, Figure S1b,d). High-resolution images in TEM reveal a narrow pore distribution with a diameter size of about 3 nm (Figure 1d). Most pores are hexagonal with compact packing.

**Figure 2:**
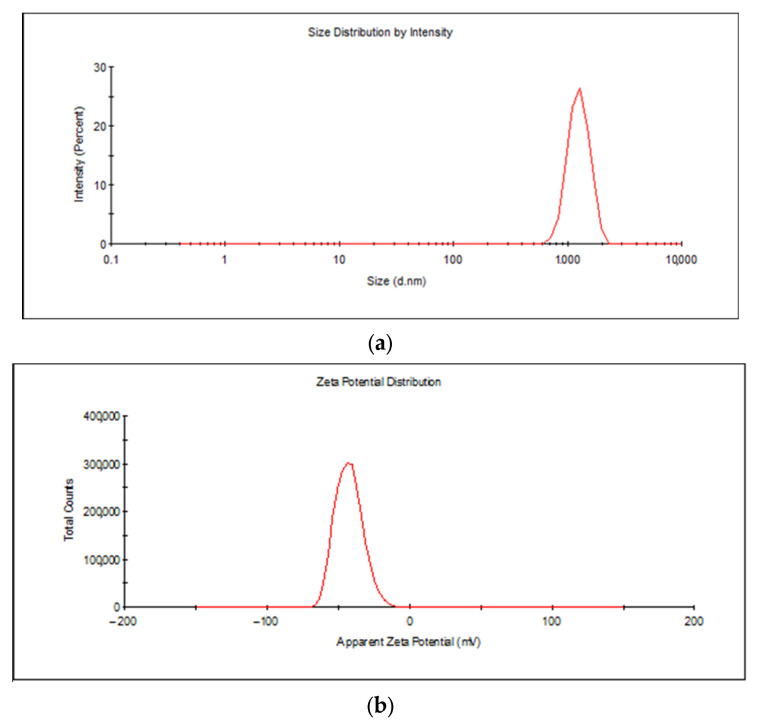
DLS analysis, size (**a**) and zeta potential (**b**) of the MCM-41 material.

According to the DLS analysis, the MCM-41 material has an average hydrodynamic diameter of 1500 nm (Figure 2a) with a polydispersity index of 0.287, which indicates good uniformity. The colloidal stability of the MCM-41-type mesoporous particles in ultrapure water (18.2 MΩ·cm) is very good according to the zeta potential of −42.5 mV (Figure 2b). The DLS data are in agreement with the results of the microscopy observations addressed in the previous paragraph.

**Figure 3:**
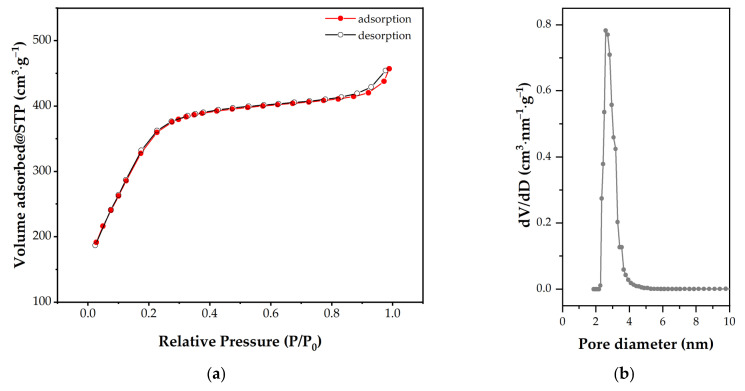
Nitrogen adsorption/desorption curves (**a**) and the pore diameter distribution (**b**) of synthesized mesoporous MCM-41-type material.

The mesoporous structure was also confirmed by the N_2_ physisorption analysis. The specific surface area of the material obtained is 1244 m^2^/g, with an average pore diameter of 2.14 nm and a total pore volume of 0.678 cm^3^/g. Also, the as-synthesized MCM-41 shows a type IV isotherm according to IUPAC standards, which is characteristic of this type of material (Figure 3a), and a uniform pore-size distribution (Figure 3b).

**Figure 4:**
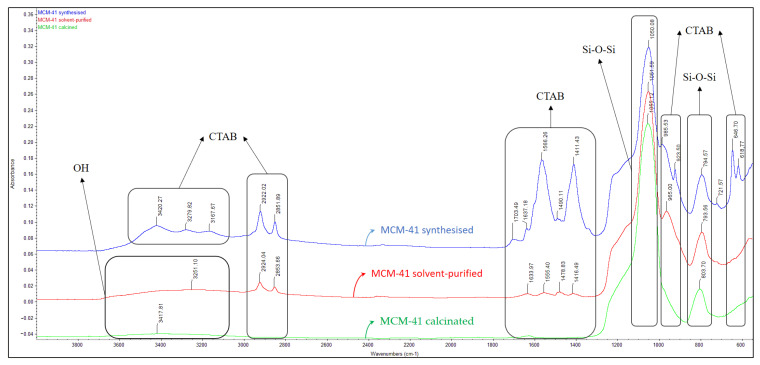
FT-IR spectra of MCM-41 as synthesized, purified by solvent extraction, and calcined materials.

In the FT-IR spectrum (Figure 4) of the synthesized MCM-41 material, the formation of the silica network is confirmed by the presence of adsorption bands specific to symmetric vibrations, 794 cm^−1^, and asymmetric vibrations, 1050 cm^−1^ (Si-O-Si).

**Figure 5:**
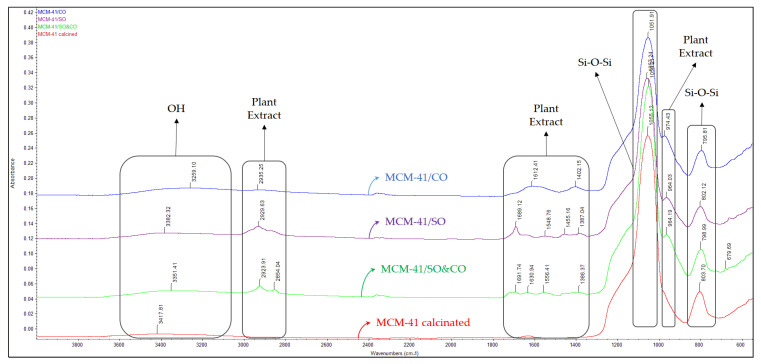
FT-IR spectra of MCM-41 calcined and loaded with plant extracts (MCM-41/SO, MCM-41/CO and MCM-41/SO&CO).

The specific bands of the template agent (CTAB) can be observed in the synthesized spectrum (Figure 5): 618 cm^−1^, 646 cm^−1^, 923 cm^−1^, 985 cm^−1^, 1411 cm^−1^, 1490 cm^−1^, 1566 cm^−1^, 1637 cm^−1^, 1703 cm^−1^, 2851 cm^−1^, 2922 cm^−1^, 3167 cm^−1^, 3279 cm^−1^ and 3420 cm^−1^. After the first extraction purification step by using ethanol extraction, some of the specific CTAB bands disappeared and others decreased in intensity: 1416 cm^−1^, 1478 cm^−1^, 1555 cm^−1^, 1633 cm^−1^, 2853 cm^−1^ and 2924 cm^−1^. Following the calcination process, the elimination of the specific bands of the template agent can be observed, meaning that the organic compound was completely removed from the pores of the mesoporous material. In the calcinated material spectrum remained the specific bands of the MCM-41-type mesoporous material: 3417 cm^−1^, characteristic of the -O-H stretching vibrations corresponding to the water molecules adsorbed in the matrix, which overlapped with the -O-H stretching vibrations, characteristic of the vicinal silanol groups associated with hydrogen bonds; 1055 cm^−1^, characteristic of the asymmetric stretching of -Si-O-Si- groups; and a broad band at 803 cm^−1^, characteristic of the symmetric stretching of -Si-O-Si- groups.

The comparative results analysis proves the complete surfactant removal after the purification process [44].

### 3.2. Loading Process of Bioactive Extracts on Mesoporous Matrix 

The FT-IR spectra of the new composites show the presence of the bands specific to the compounds of the extracts: 974 cm^−1^, 1402 cm^−1^, 1612 cm^−1^ and 2935 cm^−1^ for MCM-41/CO; 964 cm^−1^, 1387 cm^−1^, 1455 cm^−1^, 1548 cm^−1^, 1689 cm^−1^ and 2929 cm^−1^ for MCM-41/SO; and 964 cm^−1^, 1386 cm^−1^, 1556 cm^−1^, 1630 cm^−1^, 1691 cm^−1^, 2854 cm^−1^ and 2923 cm^−1^ for MCM-41/SO&CO (Figure 5). They confirm the loading process with the two extracts.

### 3.3. Quantification of Bioactive Compounds and Release Profiles of the MCM-41/SO&CO Composite Material

**Figure 6:**
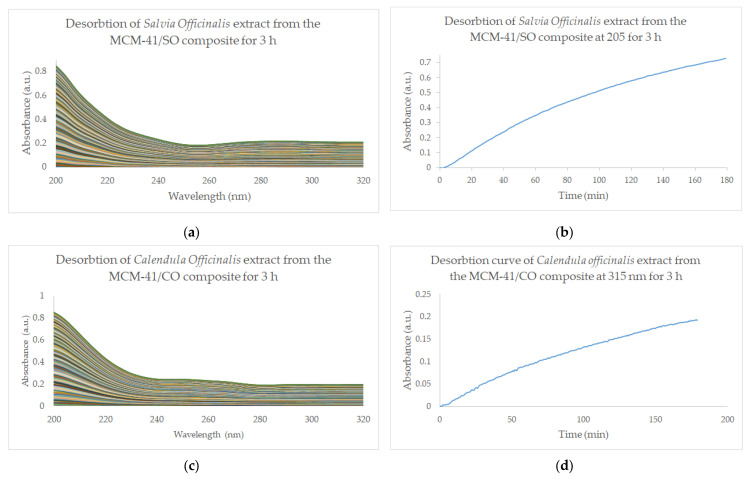
Qualitative monitoring of the desorption degree of MCM-41/SO (**a**,**b**) and MCM-41/CO (**c**,**d**) composites for 3 h. (**a**,**c**) shows the desorption kinetics of the extract from the composite represented by UV-VIS spectra acquired every 45 s.

Experiments carried out by using equipment comprising a continuous flow cell and a UV-VIS detector allow us to measure a very low concentration of bioactive compounds due to the long path through the sample = 10 mm. The acquired data (absorbance/throughout the scanned range) and the absorbance values at wavelengths specific to the bioactive complex (205 nm for triterpene acids and 315 nm for phenolic acids) were represented graphically. The qualitative monitoring of the desorption profile of the MCM-41/SO and MCM-41/CO composites showed a steady release over 3 h (Figure 6b,d).

**Figure 7:**
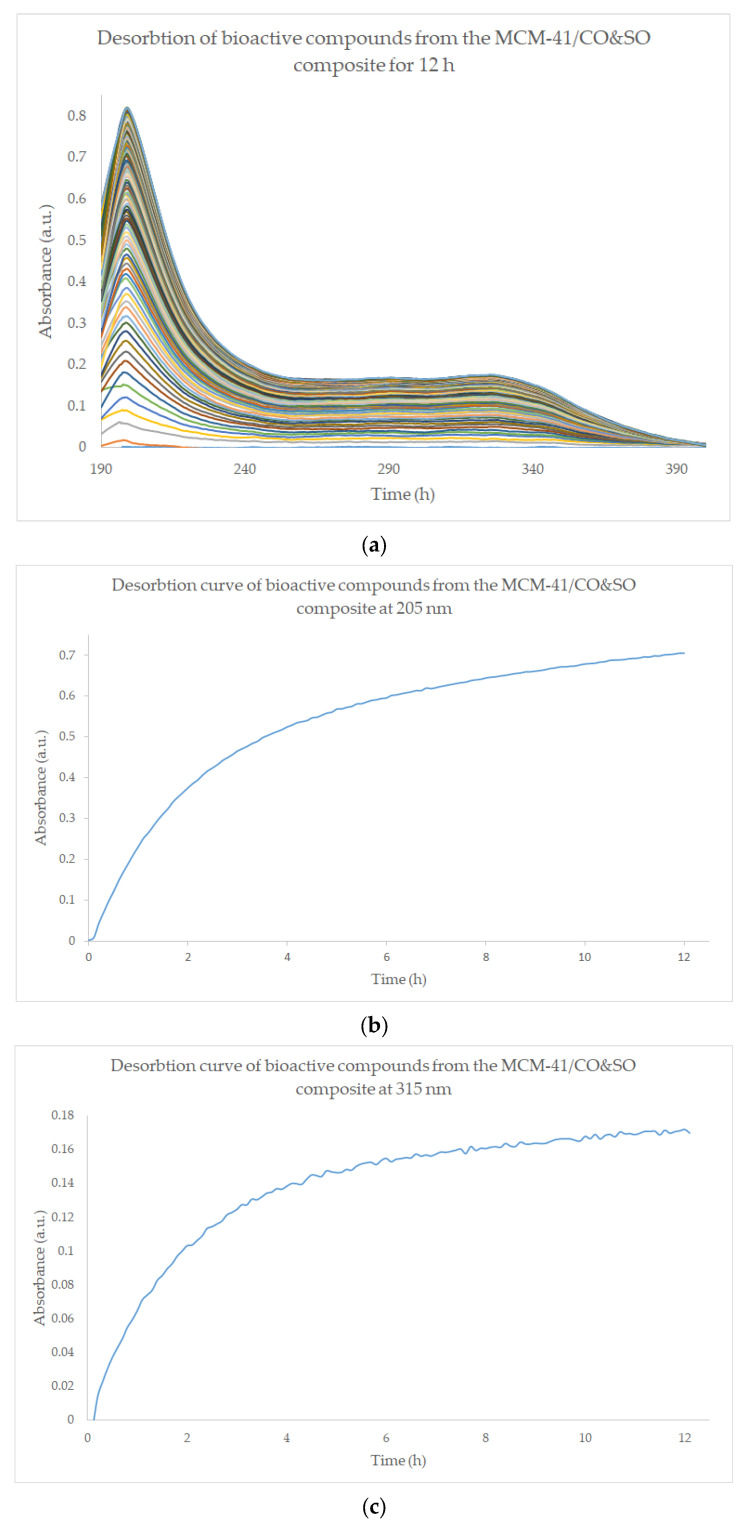
Qualitative monitoring of the desorption degree of MCM-41/SO&CO composite for 12 h (**a**–**c**). (**a**) shows the desorption kinetics of the extract from the composite represented by UV-VIS spectra acquired every 45 s.

Qualitative monitoring of the desorption of the MCM-41/SO&CO composite material revealed a significant difference in the release of bioactive compounds in the two major regions of the profile. In the first region (0–3 h), there is a rapid release from the composite surface of the bioactive compounds, while in the second region (3–12 h), the slope of the desorption curve changes significantly and the compounds are released slowly from within the pores, but in a sustained manner (Figure 7b,c).

Table 1 presents the quantitative results for the representative compounds.

**Figure 8:**
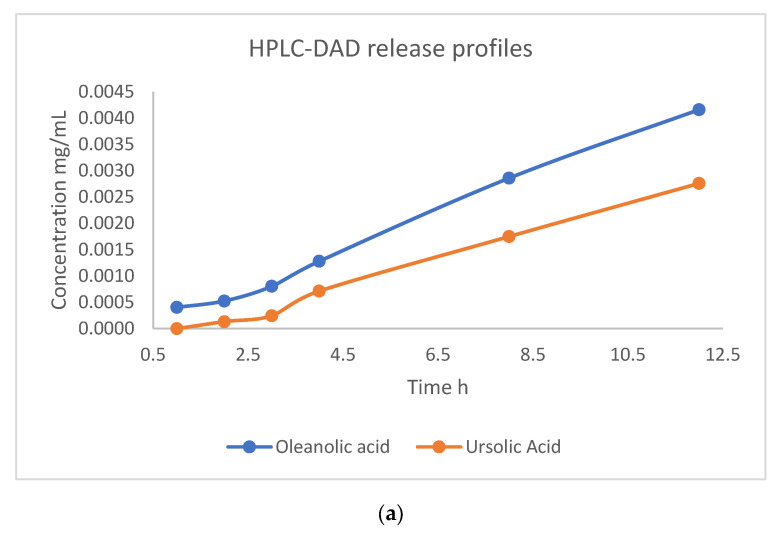

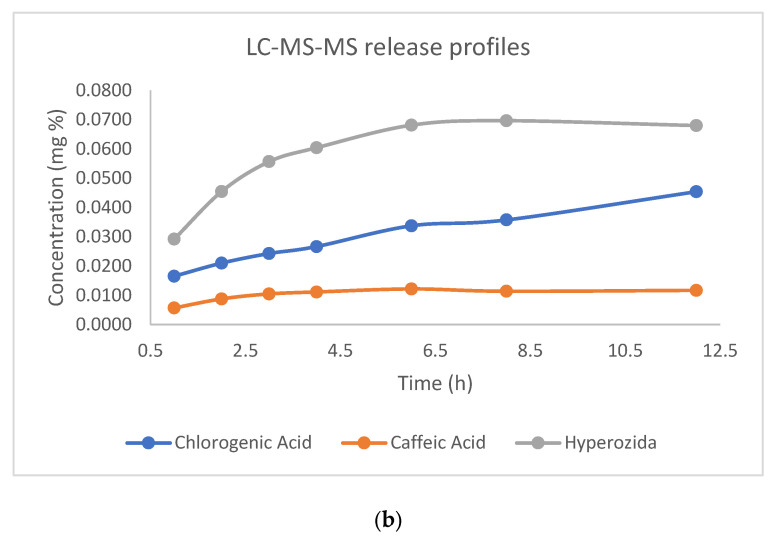
Release profiles of triterpenic acids using HPLC-DAD analysis (**a**) and the release profiles of caffeic acid, chlorogenic acid and hyperoside using LC-MS-MS analysis (**b**) for the MCM-41/SO&CO composite for 12 h.

For the quantitative determination of triterpenic acids, caffeic acid, chlorogenic acid and hyperoside, 100 mg of MCM-41/SO&CO was placed in 100 mL of phosphate-buffered saline solution, and samples were taken at predetermined intervals for the HPLC and LC-MS-MS analysis. From the release profile of the desorption study for the triterpene acids, one can observe a slow and sustained release of ursolic and oleanolic acids over 12 h, indicating that not all triterpene compounds were released from the pores of the mesoporous composite (Figure 8a and Supplementary Materials, Figure S2). From the LC-MS-MS desorption profiles, the one of hyperoside can be divided into three regions: the first region implies a rapid release in the first 3 h; in the second region (3–7 h) with a difference in slope, the speed of release is slow and sustained; and in the third region (7–12 h), the concentration remains constant, a fact that confirms the cessation of the desorption. The release profile of chlorogenic acid has a slow and sustained release throughout the whole range. Caffeic acid has a very slow release, and after 3 h, the concentration remains constant (Figure 8b and Supplementary Materials, Figure S3).

### 3.4. Evaluation of the Biological Action of Composite Materials

An investigation of the mechanisms of action was performed on the two cell lines HS27 and HaCaT that are naturally involved in tissue-regeneration processes. In order to simulate the inflammation associated with skin lesions, the unstimulated cells were compared with the proinflammatory state induced by TNF-α or PMA stimulation, respectively. The action of MCM-41, MCM-41/SO, MCM-41/CO and MCM-41/SO&CO was analyzed in comparison with the effect produced by Vitamin C, which is used in the experimental series as a positive control because of its known properties: strong antioxidant activity, stimulating synthesis and rebuilding collagen fibrils. 

**Figure 9:**
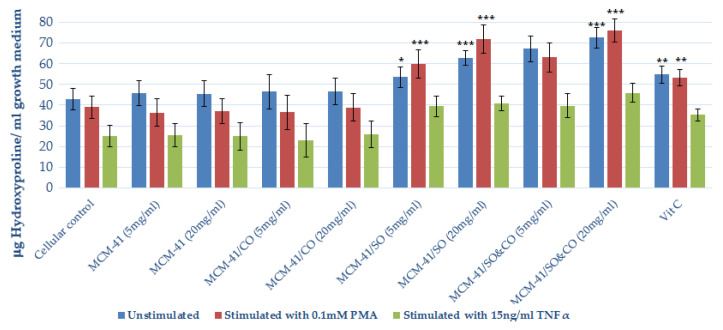
Evaluation of collagen biosynthesis process in HS27 culture under the action of MCM-41/SO, MCM-41/CO and MCM-41/SO&CO complexes. Statistical analysis was performed by applying the ANOVA method (* *p* < 0.05, ** *p* < 0.01 and *** *p* < 0.001 vs cellular marker).

A quantitative determination of the total collagen secreted by the HS27 fibroblast cell line into the extracellular medium was performed indirectly based on the hydroxyproline concentration resulting from the acid hydrolysis of collagen. The experimental results are shown in Figure 9.

From the analysis of the experimental data obtained from the application of the biochemical test models (see Figure 9), the ability of MCM-41/SO&CO to induce collagen biosynthesis in the HS27 culture after 72 h of exposure in a dose–effect manner is evident. This was observed both in experimental series performed under normal growth conditions and in those in which oxidative stress was induced by introducing the proinflammatory stimuli PMA and TNF-α into the culture. MCM-41/SO is remarkable in the experimental series carried out under normal and PMA stimulation conditions when, also in this case, collagen accumulation occurs as a function of the applied dose, starting from values comparable to the cellular control and up to 46.56% in normal conditions and 53.75% in stress conditions for the highest applied dose (20 mg/mL). MCM-41/CO showed insignificant action on the fibroblast cell line regardless of the applied dose or time of action. MCM-41 was found to be an inert material as it did not influence the biosynthesis process of collagen molecules in the extracellular matrix, with the values recorded as being statistically comparable with the cell control.

**Figure 10:**
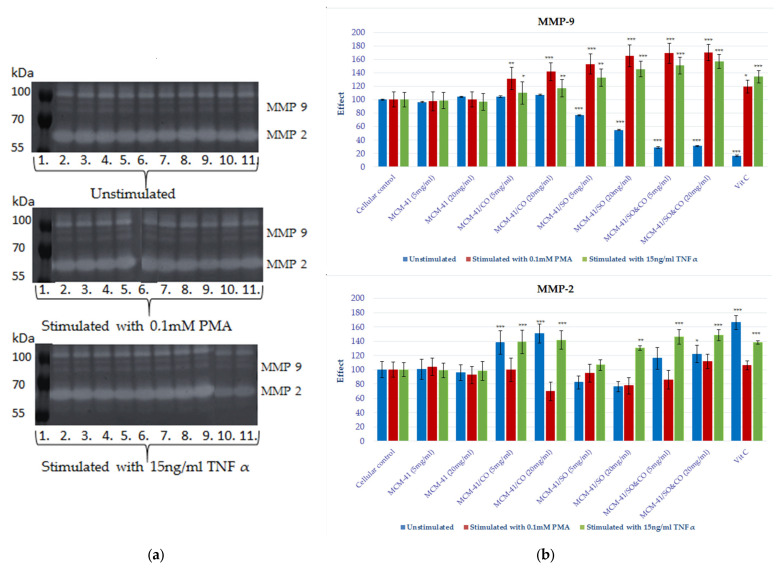
(**a**) Gelatin zymography of the medium samples collected from HS27 cell culture. Each lane represents a different compound tested, such as: 1. molecular marker; 2. cellular marker; 3. MCM-41, 5 mg/mL; 4. MCM-41, 20 mg/mL; 5. MCM-41/CO, 5 mg/mL; 6. MCM-41/CO, 20 mg/mL; 7. MCM-41/SO, 5 mg/mL; 8. MCM-41/SO, 20 mg/mL; 9. MCM-41/SO&CO, 5 mg/mL; 10. MCM-41/SO&CO, 20 mg/mL; and 11. Vitamin C, 10 µg/mL. (**b**) The graphs highlight the enzyme activity levels of MMP-9 and MMP-2. By densitometric analysis with Image J 1.48v software (https://imagej.net/ij/, accessed on 10 July 2013), the volume of the lysis band was calculated (measuring the intensity of each band and the corresponding surface area) which, in relation to the value of the volume of the cell marker, allowed for the evaluation of the effect of the test substance on the cell line. Statistical analysis was performed by applying the ANOVA method (* *p* < 0.05, ** *p* < 0.01 and *** *p* < 0.001 vs. cellular marker).

**Figure 11:**
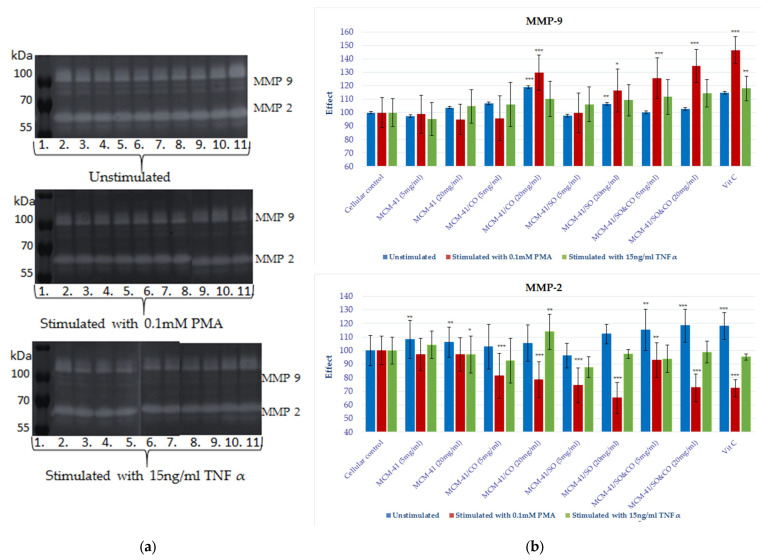
(**a**) Gelatin zymography of the medium samples collected from HaCaT cell culture. Each lane represents a different compound tested, such as: 1. molecular marker; 2. cellular marker; 3. MCM-41, 5 mg/mL; 4. MCM-41, 20 mg/mL; 5. MCM-41/CO, 5 mg/mL; 6. MCM-41/CO, 20 mg/mL; 7. MCM-41/SO, 5 mg/mL; 8. MCM-41/SO, 20 mg/mL; 9. MCM-41/SO&CO, 5 mg/mL; 10. MCM-41/SO&CO, 20 mg/mL; and 11. Vitamin C, 10 µg/mL. (**b**) The graphs highlight the enzyme activity levels of MMP-9 and MMP-2. By densitometric analysis with Image J 1.48v software (https://imagej.net/ij/, accessed on 10 July 2013), the volume of the lysis band was calculated (measuring the intensity of each band and the corresponding surface area) which, in relation to the value of the volume of the cell marker, allowed for the evaluation of the effect of the test substance on the cell line. Statistical analysis was performed by applying the ANOVA method (* *p* < 0.05, ** *p* < 0.01 and *** *p* < 0.001 vs. cellular marker).

It is well known that collagen degradation in the extracellular matrix is mainly due to the proteolytic activity of metalloproteinases that are expressed by both dermal (fibroblasts and keratinocyte) and inflammatory cells, and these enzymes have an important role in the matrix remodeling of skin subjected to a prolonged inflammatory process [45,46]. The synthesis and secretion of MMPs occur in the presence of cellular inflammation and are a clear indicator of this process. MMP2 and MMP9 are the two gelatinases that are known for their activities in destroying the matrix around cells, facilitating the access of various factors to target cells. These matrix enzymes’ biosynthesis processes are stimulated by cytokines (IL1b, TNF-alpha) and also by compounds such as lipopolysaccharides (LPSs) or PMA. The use of TNF and PMA as proinflammatory stimuli on fibroblast and keratinocyte cell cultures allowed us to evaluate the effect of the bioactive compound release system of the mesoporous composite on the proteolytic activity of the matrix gelatinases involved in tissue-wound-healing processes (see Figure 10 and Figure 11).

After the analysis of the zymograms obtained by electrophoretic migration in gelatin-copolymerized polyacrylamide gel, the enzymatic activity of MMP9 is increased both under basal and nonspecific proinflammatory stimulation conditions on both of the tested HS27 and HaCaT lines. The most remarkable finding from the systems evaluated came from MCM-41/SO&CO, which, on both cellular lines, potentiated MMP9 activity in a dose–effect manner under conditions of proinflammatory stimulation. After a 72 h treatment of fibroblast cell lines with MCM-41/SO&CO, the MMP9 activity increased by 70% under PMA stimulation and by 57% under TNF-alpha stimulation, and in the same conditions, proteolytic activation on the HaCaT line was 34% for the PMA stimulation and 14% for oxidative stress induction with TNF-alpha. This behavior is also observed in MCM-41/CO- and MCM-41/SO-treated cells, but with a lower expression.

**Figure 12:**
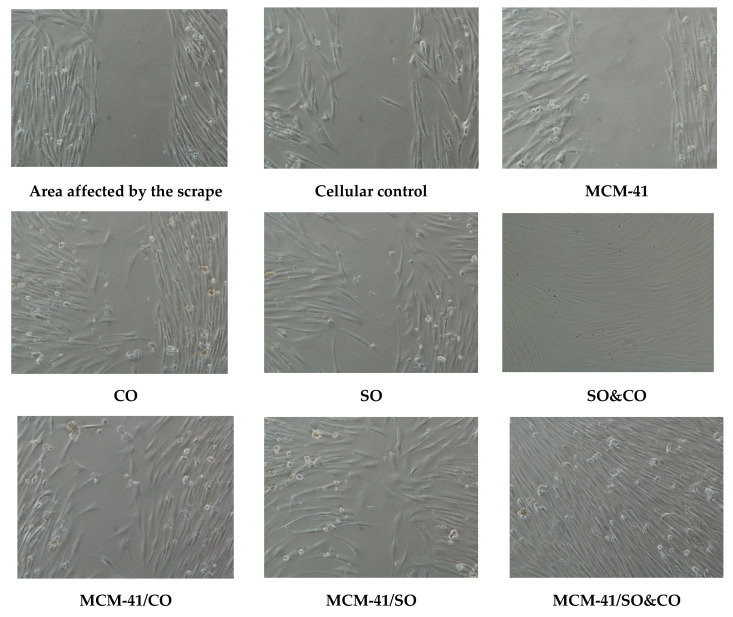
Two-dimensional method for assessing the migration rate of fibroblast cells (HS27) at the wound site under the action of 20 mg/mL mesoporous material (MCM-41, MCM-41/CO, MCM-41/SO and MCM-41/SO&CO) and extract added in the amount equivalent to the loaded in the MCM-41 type mesoporous material, respectively 6mg/ml (CO, SO and SO&CO). The presented images were acquired at 24 h after the formation of the scratch on the monolayer.

Cell migration is a multistep process depending on the composition of the extracellular matrix and involving changes in cytoskeletal organization, cell–cell/substrate–cell adhesion and other specific changes in associated cellular components, playing an important role in a variety of pathophysiological processes such as wound healing, tumor invasion and metastasis [46]. In wound healing, the process by which epithelial cells mi-grate, replicate and cross the wound is through mitosis, which is the predominant mechanism. Figure 12 shows the images acquired during the test to evaluate the migration process of HS27 fibroblast cells, which involved a wound simulation by scratching the cell monolayer that reached the confluence. An analysis of the images acquired with the Nikon Eclipse Ti-S microscope and processed with Image J 1.48v software (https://imagej.net/ij/, accessed on 10 July 2013), revealed that after 24 h, MCM-41/SO&CO promotes cell migration depending on the applied dose, covering 100% of the migration distance.

## 4. Discussion

The therapeutic approach to chronic wounds is based on the etiology of the lesion and includes a series of measures (such as maintaining perfect local hygiene, a proper diet, a correction of venous stasis, peripheral ischemia and any associated metabolic deficits) that make this process an extremely difficult task [47,48,49]. It is recommended that palliative treatment and the initiation of a specific therapy be started as close as possible to the time of the injury in order to ensure a clean environment [47,48,49]. Currently, the application of bandages with some regularity is the most widely used therapy for wound care due to their properties as wound-healing promoters (such as collagen containing scaffolds that promote cell migration, facilitating organized cicatrization) and low costs [47,48,49]. In current practice, a wide range of products with different properties are used, making them suitable for one type of wound and not suitable for others because of their difficult removal process or their high cytotoxicity. Taking into account the need to diversify therapeutic approaches, the potential delivery systems and the bioactive compounds isolated from natural sources, several new R&D strategies have been initiated and numerous emerging technologies have been explored, with the aim of obtaining smart therapeutic solutions that can ensure an efficient healing process. Thus, due to their biocompatibility, biodegradability and ability to absorb molecules, the Poly-L-Lactic Acid (PLLA) biopolymer has become the first choice in the development of delivery systems for active pharmaceutical ingredients [47,48,49]. In the present case, we aimed to obtain an efficient delivery system based on MCM-41-type mesoporous material for bioactive extracts rich in polyphenols, flavones, ursolic acid and oleanolic acid that would be biocompatible and nontoxic to create a microenvironment favorable to accelerate the healing process by inducing the extracellular secretion of matrix biomolecules and stimulating the migration of epidermal cells into the wound site. This approach came from the need to have a product that could allow for the controlled release of the compounds loaded in the biomaterial over a long period of time and where the desorption process is not affected by the microenvironment, this being performed only depending on the physicochemical properties of the bioactive substances migrating through the pore. By loading extracts rich in polyphenols, flavones, ursolic acid and oleanolic acid into MCM-41-type mesoporous material, the bioavailability, light stability (e.g., polyphenols are easily oxidized by exposure to light) and the concentration of active substances that can be introduced into the pharmaceutical formulation is increased (very often, due to low solubility, the concentrations of compounds that are effective in in vitro tests cannot be used in in vivo studies) [47,48,49].

The two-step profile of the tested composite is based on the initial rapid desorption of bioactive compounds to achieve a fast and effective therapeutic concentration upon application; this occurs due to the bioactive compounds present on the surface of the composite, whereby the long-term, slow desorption of bioactive compounds occurs and they get adsorbed inside the pores of the mesoporous material.

The evaluation of the stimulating properties of the MCM-41, MCM-41/SO, MCM-41/CO and MCM-41/SO&CO was carried out by conducting a correlation analysis on the actions involved in this process: collagen biosynthesis, the activity of proteolytic enzymes such as metalloproteinases and cell migration in the wound site. At various stages of the wound-healing process, an essential role is attributed to matrix metalloproteinases (such as collagenases and gelatinases) with proteolytic activity on both intact and damaged fibrillar collagen, which guide the remodeling of repaired tissue by regenerating collagen molecules from the extracellular matrix. The synthesis and release of these enzymes are highly regulated and associated with specific cell subtypes [50,51]. The matrix metalloproteinase (MMP) family consists of 28 structurally related Zn-dependent metalloendopeptidases secreted or overexpressed on the cell surface that mediate the pH-neutral hydrolysis of structural molecules in the extracellular matrix, both in vivo and in vitro, and are involved in the inflammatory mediator and growth-factor processes [52]. Because of these properties, this group of enzymes is very important in tissue remodeling and wound healing, but they are also an important element in the progression of inflammatory diseases [53]. They are secreted in latent forms (proMMPs) that are activated in the extracellular space by the cleavage of an N-terminal propeptide [52,53]. In human skin, a number of MMPs expressed by keratinocytes have been shown to be activated during the reepithelization process after wound healing as well as in some dermatoses (dermal fibrosis and melanoma) [52,53]. The regulation of the extracellular matrix involves a balance between the synthesis and degradation of the structural components under the catalytic action of MMPs. Collagen, a ubiquitous component and the most abundant protein in the human body (30% of the total protein mass), is a biopolymer with an open internal structure containing a large number of binding sites available for interactions with different biologically active substances such as hormones, enzymes, peptides, etc. All these interactions, facilitated by its extraordinary hydrophilic capacity, give the collagen molecule (both in its fibrillar conformation and in the form of soluble collagen polypeptides) an extremely important role in the regulation of wound-healing phases. In addition to its structural role, which confers elasticity to the skin, collagen participates in the initiation and unfolding of some complex developmental, morphogenesis and pathological processes, being directly and/or indirectly involved in cell attachment and differentiation as well as immunological processes [41,50,51,52,54]. A correlative analysis of the experimental results revealed that MCM-41 is an inert material that does not affect the epithelial cells both in terms of migration speed into the wound site and metabolic processes; additionally, collagen biosynthesis and the proteolytic action of the metalloproteinase (MMP-2 and MMP-9) released into the extracellular matrix are comparable to that recorded for cellular control. The mesoporous compound loaded with a single plant extract applied at a dose of 20 mg/mL increased the migration rate by approximately 30% for MCM-41/CO and 60% for MCM-41/SO. On the other hand, the same silica matrix loaded with both extracts (MCM-41/SO&CO) promotes cell migration, according to the applied dose, and covers 100% of the migration distance after 24 h, which suggests a synergistic action of the plant bioactive compounds contained in the charged plant extracts. At the same time, the experimental data revealed that by incorporating the extracts into the pores of the material, their effect on the rate of cell migration into the wound site (scratch test) is not affected (Figure 12).

## 5. Conclusions

The present study describes the synthesis of mesoporous MCM-41-type material under normal temperature and pressure conditions by using sodium trisilicate as a silica source, thus significantly reducing the synthesis costs. The material was purified by using ethanol extraction followed by the calcination stage, a step confirmed by FT-IR-ATR analysis. The morphology and ordered pore structure of the mesoporous material were confirmed by SEM and TEM microscopy and the pore distribution. A N_2_ absorption/desorption analysis shows a very high specific surface area, 1244 m^2^/g, and average pore diameter, 2.14 nm, obtained after synthesizing the material. The DLS analysis shows a uniformity in the size of the MCM-41 particles of approximately 1500 nm. All these data indicate that the desired material can be used in a system involving the prolonged release of bioactive compounds for therapeutic purposes. By loading the material, MCM-41, with sage and marigold extracts, new composite materials with novel properties were obtained; this was confirmed by an FT-IR analysis, where the specific bands of the two extracts were highlighted.

Desorption profiles are a key parameter to evaluate the behavior of the composites in terms of the sustained desorption of bioactive compounds, related to maintaining an effective long-term therapeutic concentration for skin applications.

From the desorption studies carried out by using UV-VIS, HPLC and LC-MS-MS detection systems, two-step profiles were obtained for wound treatment: an initial rapid desorption of bioactive compounds for the rapid achievement of an effective therapeutic concentration for applications due to the bioactive compounds present on the surface of the composites, and a long-term slow desorption of the bioactive compounds adsorbed inside the pores of the mesoporous material.

All mesoporous silica-based composites induced, in a dose–effect manner, collagen biosynthesis; enhanced the enzymatic activity of the main metalloproteinases (MMP2 and MMP9) involved in tissue-remodeling processes; and stimulated cell migration at the wound site. Considering all these re-epithelizing properties experimentally observed on the two standardized cell lines (HS27 and HaCaT), it can be concluded that these structures can be used as main components in the development of topical products with great potential in wound healing.

## Figures and Tables

**Table 1:** The content of representative bioactive compounds in MCM-41/SO&CO.

Compound Name	Concentration (g %)
Oleanolic acid	2.41
Ursolic acid	2.15
Caffeic acid	0.0013
Chlorogenic acid	0.0084
Hyperoside	0.0104

## Data Availability

Not applicable.

## Supplementary Materials

The following supporting information can be downloaded at https://www.mdpi.com/article/10.3390/pharmaceutics15092258/s1: Figure S1: SEM images (a,b) and TEM images (c,d) at different magnifications taken of the calcined mesoporous material of type MCM-41; Figure S2: HPLC-DAD release-profile chromatograms of ursolic and oleanolic acid for 12 h; Figure S3: LC-MS-MS release-profile chromatograms of chlorogenic acid (gray peaks), caffeic acid (blue peaks) and hyperoside (green peaks) for 12 h.
