# Supplementary material for: Mesoporous Composite Bioactive Compound Delivery System for Wound-Healing Processes
Source: Pharmaceutics. 2023 Aug 31;15(9):2258. doi: 10.3390/pharmaceutics15092258 (PMC10534662; doi:10.3390/pharmaceutics15092258)
Supplement: Supplementary file 1 [file pharmaceutics-15-02258-s001.zip › pharmaceutics-2564240-supplementary.pdf]

Supplementary Material

Mesoporous composite bioactive compound delivery system for wound healing processes

Bogdan Purcăreanu[1,2], Manuela Diana Ene[1], Alina Moroșan[3], Dan Eduard Mihaiescu[3], Mihai Alexandru Florea[1], Adelina Ghica[1], Roxana Andreea Niță[1], Veronica Drumea[1], Mihai Alexandru Grigoroșcuță[4], Andrei Kuncser[4], Petre Bădică[4] and Laura Olariu[1,5]

1 Biotehnos SA, Gorunului Street 3-5, 075100 Otopeni, Romania; bogdan.purcareanu@biotehnos.com (B.P.); diana.ene@biotehnos.com (M.D.E.);alexandru.florea@biotehnos.com (M.A.F.); adelina.bicu@biotehnos.com (A.G.); rnita@biotehnos.com (R.A.N.); veronica.drumea@biotehnos.com (V.D.); lolariu@biotehnos.com (L.O.);

2 Department of Science and Engineering of Oxide Materials and Nanomaterials, Faculty of Chemical Engineering and Biotechnologies, University POLITEHNICA of Bucharest, Gh. Polizu 1-7, 011061 Bucharest, Romania; bogdanpb89@gmail.com (B.P.)

3 Department of Organic Chemistry "Costin Nenițescu", Faculty of Chemical Engineering and Biotechnologies, University POLITEHNICA of Bucharest, Gh. Polizu 1-7, 011061 Bucharest, Romania; alina.morosan@upb.ro (A.M.); dan.mihaiescu@upb.ro; danedmih@gmail.com (D.E.M.);

4 National Institute of Materials Physics, Street Atomistilor 405 A, 077125 Magurele, Romania; alex_bebe07@yahoo.com (M.A.G.); akuncser@yahoo.com (A.K.); badica2003@yahoo.com (P.B.)

5 Academy of Romanian Scientists, 3 Ilfov Street, 030167 Bucharest, Romania

* Correspondence: dan.mihaiescu@upb.ro; danedmih@gmail.com (D.E.M.); diana.ene@biotehnos.com (M.D.E.);

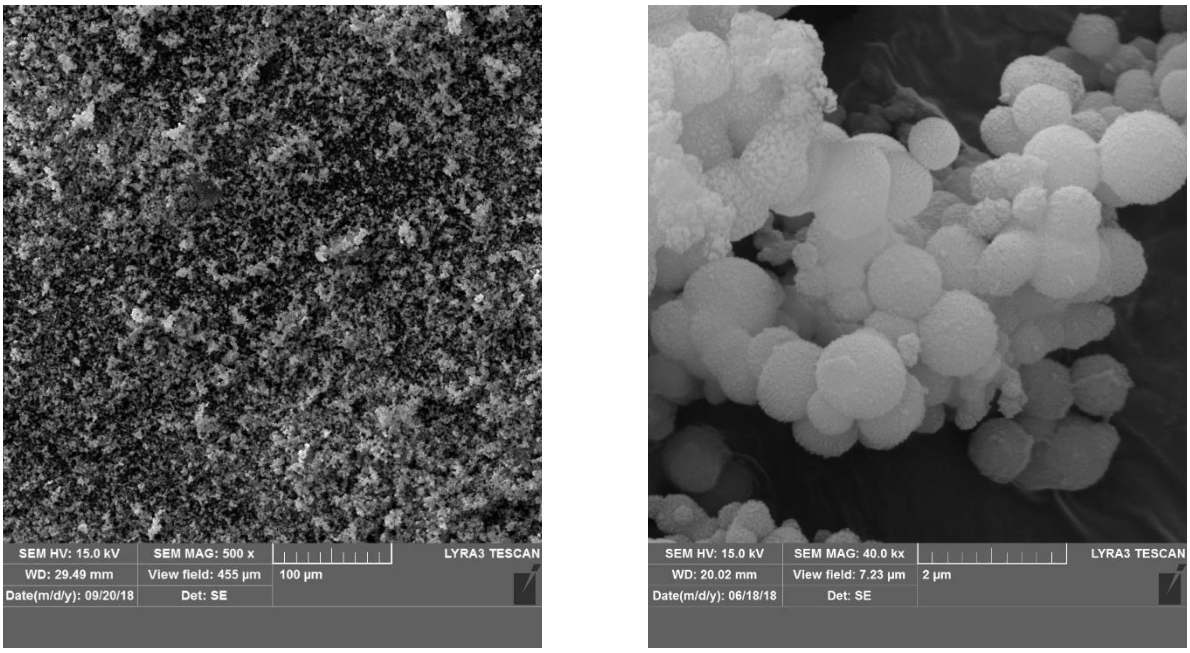

(**a**) (**b**)

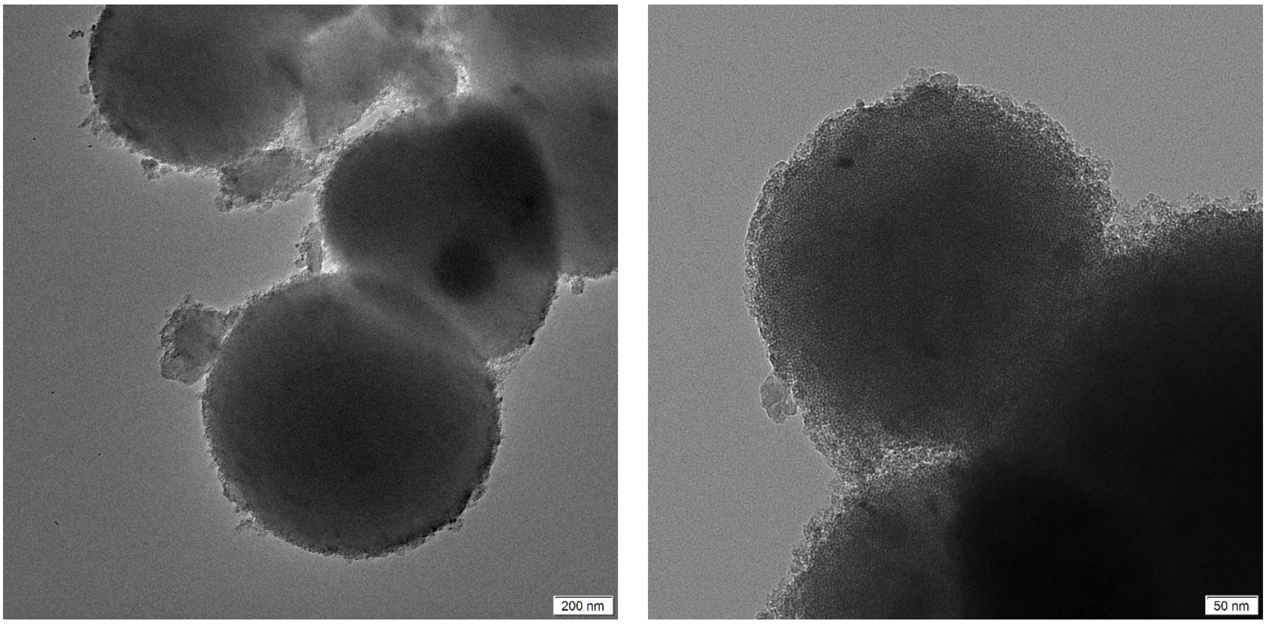

(**c**) (**d**)

Figure S1. SEM images (a - b) and TEM images (c - d) at different magnifications taken on the calcined mesoporous material of type MCM-41.

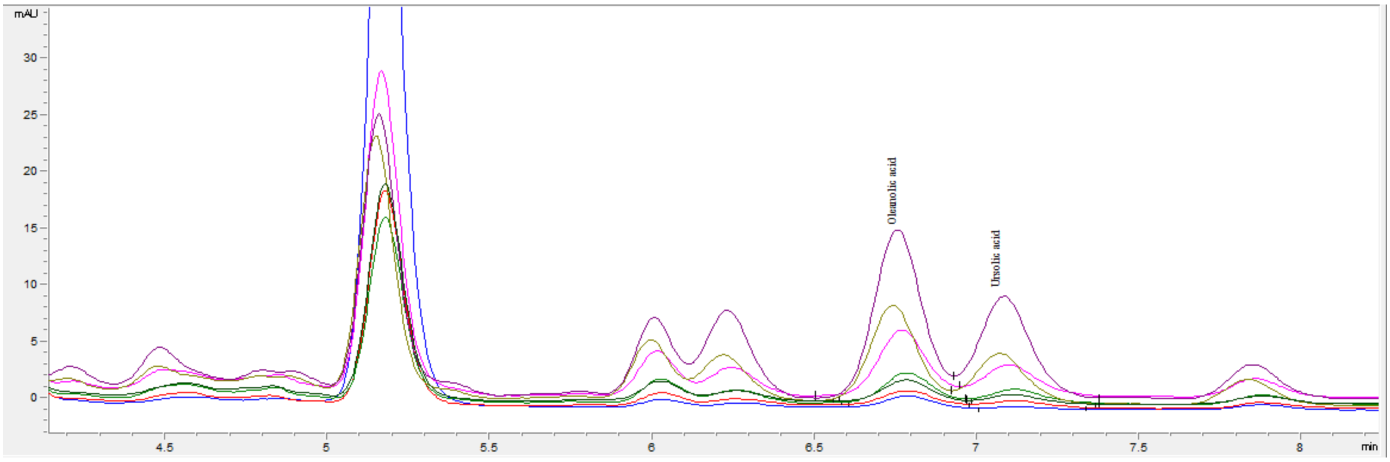

Figure S2. HPLC-DAD release profile chromatograms for ursolic and oleanolic acid for 12 h

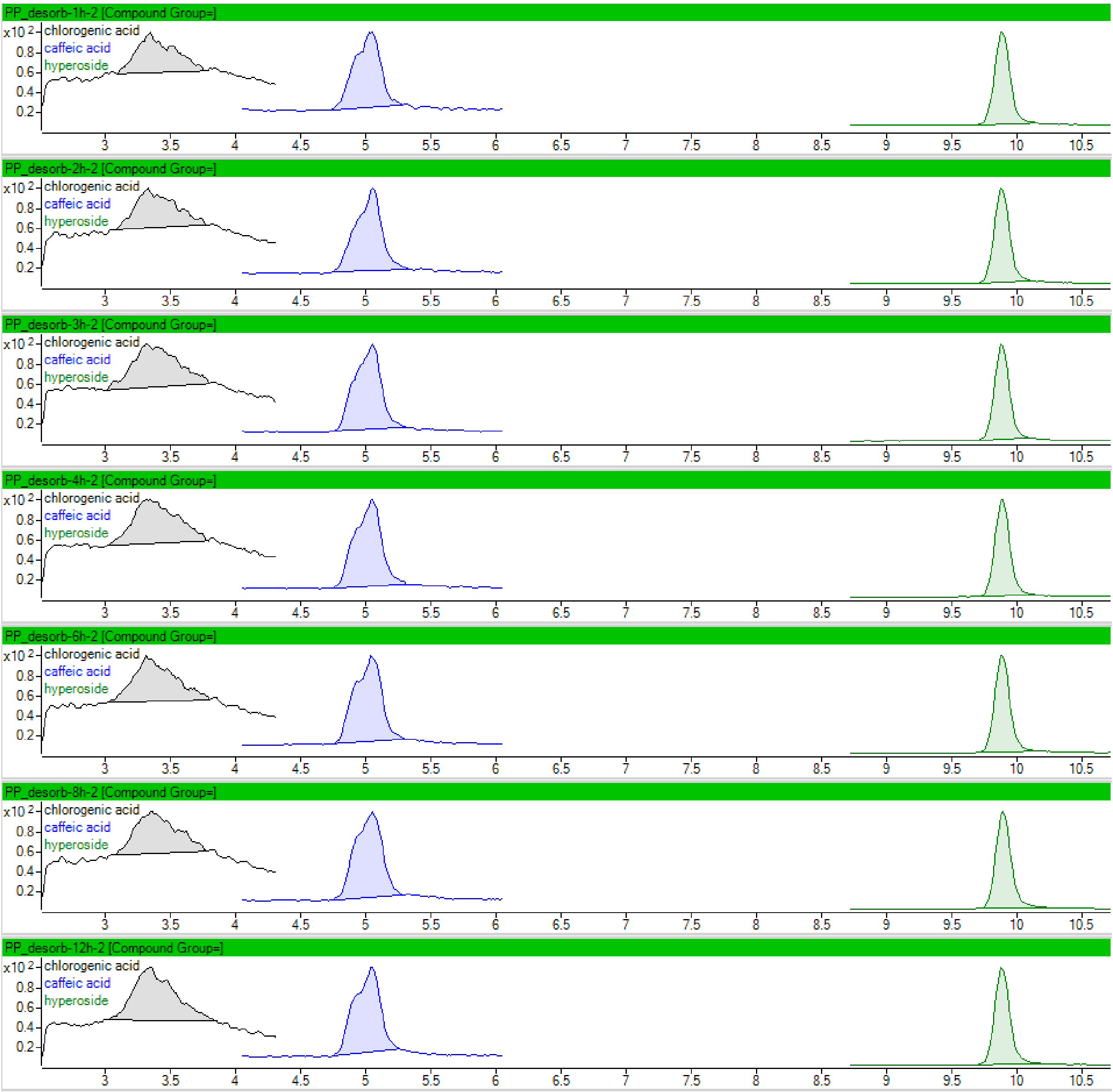

Figure S3. LC-MS-MS release profile chromatograms for chlorogenic acid (gray peaks), caffeic acid (blue peaks), and hyperoside (green peaks) for 12 h
